# Evaluating the global prevalence of insomnia during pregnancy through standardized questionnaires and diagnostic criteria: a systematic review and meta-analysis

**DOI:** 10.3389/fpsyt.2024.1427255

**Published:** 2024-08-13

**Authors:** Chengcheng Yang, Rui Fu, Huan Wang, Yanjie Jiang, Shipeng Zhang, Xiaoli Ji

**Affiliations:** ^1^ Hospital of Chengdu University of Traditional Chinese Medicine, Chengdu University of Traditional Chinese Medicine, Chengdu, China; ^2^ Nanjing University of Chinese Medicine, Nanjing, China

**Keywords:** insomnia, depression, geographic location, prevalence during pregnancy, global

## Abstract

**Introduction:**

Insomnia during pregnancy presents significant medical care challenges and heightens the risk of adverse outcomes for both pregnant women and fetuses. This study undertook a meta-analysis to assess the global prevalence of insomnia during pregnancy, examining both the overall prevalence and regional variations.

**Method:**

The aim of this study was to perform a meta-analysis of articles indexed in PubMed, Embase, and Web of Science from the inception of these databases up to February 29, 2024. The study systematically reviewed the global prevalence of gestational insomnia and explored potential moderating factors, encompassing research type, publication date, regional influences, maternal age, pregnancy status, depressive symptoms, and anxiety symptoms.

**Result:**

Forty-four studies, encompassing a total of 47,399,513 participants, were included in the analysis. The overall prevalence of insomnia symptoms during pregnancy was 43.9%. Regional factors and depression emerged as the main factors affecting insomnia, with Europe (53.6%) surpassing North America (41.0%), followed by South America (50.6%) and Asia (40.7%). High depression rates (56.2%) correlated with increased insomnia prevalence compared to low depression rates (39.8%). The type of research and publication date showed no significant impact on the prevalence of insomnia symptoms.

**Conclusion:**

The meta-analysis results indicated that the prevalence of insomnia symptoms was higher during pregnancy, especially among pregnant women who were in a highly depressed state or located in the European region.

**Systematic review registration:**

PROSPERO, identifier CRD42018104460.

## Highlight

- Reassess the global prevalence of insomnia during pregnancy.
- The regional differences in insomnia disease were discussed.
- The possible reasons for the difference in prevalence were analyzed.

## Introduction

Insomnia is a common clinical disorder characterized by difficulty falling asleep or maintaining sleep, often accompanied by symptoms such as irritability or fatigue when awake. It often occurs at least three times a week and lasted for a duration of at least three months, and can’t be attributed to other diseases or substances (1).

Research indicates that the incidence rate of insomnia in adults typically ranges from 6% to 10%, whereas among pregnant women it is notably higher at 38.2% (2, 3). Insomnia during pregnancy can be attributed to a varied of factors, such as physical discomfort, hormonal fluctuations, fetal growth (4), and emotional distress. Research has shown that insomnia during pregnancy (5) not only leads to a decline in quality of life, but also is a potential cause of premature delivery, cesarean section, prolonged delivery, pregnancy induced hypertension, pregnancy induced diabetes, and postpartum depression. Lu conducted a comprehensive review of sleep disorders and their association with adverse maternal and infant outcomes. The results revealed that sleep disorders, including insomnia, were associated with an increased risk of adverse pregnancy outcomes such as preeclampsia (OR=2.80, 95% CI 2.38-3.30), hypertension during pregnancy (OR=1.74, 95% CI 1.54-1.97), diabetes during pregnancy (OR=1.59, 95% CI 1.45-1.76), cesarean section (OR=1.47, 95% CI 1.31-1.64), and premature delivery (OR=1.38, 95% CI 1.26-1.51) (6); Additionally, a study demonstrated that insomnia was associated with an increased risk of perinatal suicide (OR=4.76, 95% CI 1.83-12.34) (7). Therefore, insomnia poses a significant threat to maternal and fetal health throughout pregnancy.

In the 2020 review on the prevalence of insomnia, the authors conducted subgroup analysis on variables including maternal age, gestational age, depressive symptoms, and gestational period (8). The review found that maternal age, gestational age, and anxiety significantly impact the prevalence of insomnia. Due to the limited number of articles included, the authors did not compare regional prevalence rates. As is well known, insomnia during pregnancy is a sleep disorder caused by various confounding factors. Regional studies are important for the prevention of women’s health. In China, a cross-sectional study on insomnia in Chinese pregnant women showed that 24.3% of the pregnant women suffered from insomnia, and found that maternal age, attained education, occupation, monthly household income, insurance coverage, relationship with the mother-in-law, gestational age, and anxiety symptoms were independently risk factors for insomnia (9). A Canadian study found that good social attention and partnership can reduce the incidence of prenatal depression and thus reduce the risk of insomnia during pregnancy (10). Similarly, several studies of pregnancy insomnia in the United States have found that degree of social concern is related to differences in the risk of pregnancy insomnia (11, 12). What’s more, different countries and regions may also have significant differences in the prevalence of insomnia during pregnancy due to diet, social life pressure, cultural background, and national women’s policies.

Based on this, we conducted a comprehensive database search and compare the prevalence of insomnia during pregnancy in different countries by region. These results will provide some reference evidence for the formulation of prevention and health policies in high prevalence areas, thereby increasing social attention to women’s health.

## Methods

A systematic review and meta-analysis were conducted on articles related to insomnia symptoms during pregnancy. Both systematic reviews and meta-analyses were reported in accordance with the PRISMA Declaration Guidelines (13). This review has been registered in the PROSPERO database (registration number: CRD42018104460). The PRISMA checklist could be found in **Supplementary Material 1**. The PICOS method was used in formulating research questions (14).

### Search strategy

A full-text search was conducted on the PubMed, Embase, and Web of Science databases, with a time limit for papers published until February 29, 2024, including articles that reported insomnia rates during pregnancy through self-report or questionnaire surveys. The search algorithm was based on terms such as ‘pregnancy’ and ‘insomnia’. Specific search strategies were in **Supplementary Material 2**.

**Figure 1:**
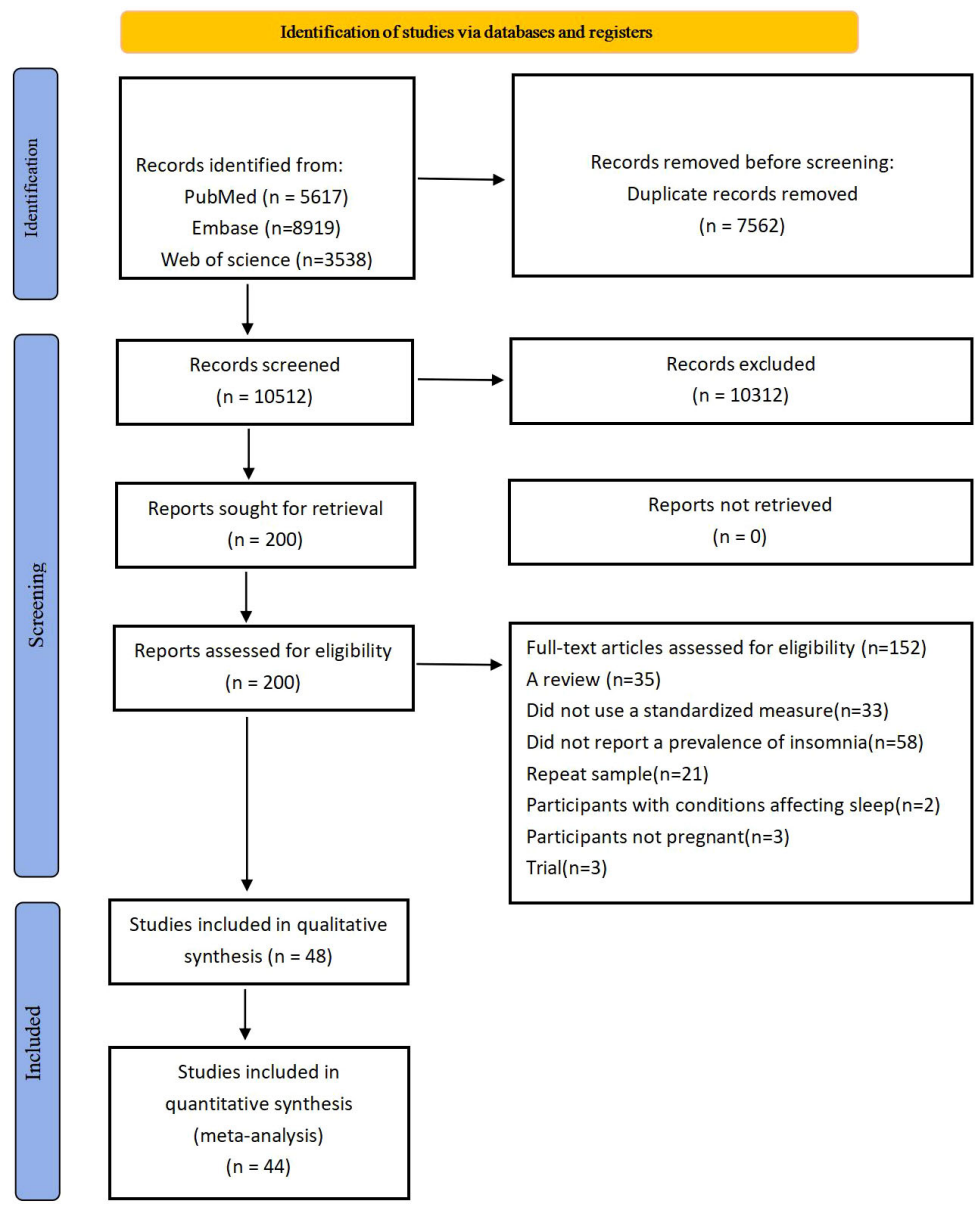
Flow chart.

The selected titles and relevant abstracts of the articles were reviewed. Each article was categorized as ‘yes’, ‘no’, or ‘possible’, with articles marked as ‘no’ being excluded from the analysis. The entire articles with titles or abstracts marked as ‘yes’ or ‘possible’ were thoroughly reviewed to determine if they met the inclusion criteria. Please refer to **Figure 1** for a detailed flowchart outlining the detection program for the study.

### Research selection

The P-population was Women with pregnancy, the I-study has no intervention, the C-compare with and without sleep problem, the O-outcome is insomnia, and S-study included Randomized controlled studies, cohort studies, cross-sectional studies, case-control studies. The study included data on the prevalence of insomnia among pregnant women, or reported the number of individuals with insomnia. Research methods involved self-reporting, questionnaire surveys, and the measurement of epidemiological data.

Exclusion criteria: The sample consisted solely of pregnant women with sleep disorders. Studies that used non-standardized measurement methods, such as evaluating insomnia with a single question, were not included. Furthermore, studies that were case reports, systematic reviews, or used data from previous studies were also excluded.

The first and second authors (FR and WH) independently reviewed these studies and conducted a full-text review based on inclusion and exclusion criteria to further exclude studies that were not qualified. Any differences were be resolved by the senior author (ZS).

### Data extraction

The first (FR) and second (WH) authors independently extracted data to confirm accuracy. The third author (ZS) confirmed the accuracy of the included data. The studies selected for review are recorded using standardized tables to describe the important variables of each study. The estimated prevalence of insomnia was obtained by extracting data on the number of cases, total sample size, or percentage of samples identified as having insomnia symptoms, as well as the study type, region, maternal age (year), gestational age (week), and percentage of anxiety or depression cases (see **Table 1**).

**Table 1:** Basic information included in the literature.

Author	Text Type	Release year	Country	Average age	Age range	Number of people	Case	Diagnostic criteria	Types of psychological problems
Mourady	Cross-sectiona	2017	Lebanon	30.52±5.22	/	141	36	Insomnia Severity Index	Depression
Palagini	Cross-sectiona	2019	Italy	33.6 ± 3	/	62	17	Insomnia Severity Index	Anxiety、Depression
David A Kalmbach	Cross-sectiona	2021	America	29.76±4.72	/	267	156	Insomnia Severity Index	Depression
Leslie M Swanson	Cross-sectional	2011	America	31	/	114	51	Insomnia Severity Index	Anxiety、Depression
Xu Chen	Cross-sectional	2023	China	29.4±4.5	/	535	320	Insomnia Severity Index	Anxiety
Xu Zhou	case–control study	2023	China	29.7±4.2	/	456	291	Pittsburgh Sleep Quality Index	/
Serap Öztürk Altınayak	Cross-sectional	2023	Turkey	27.4±6.3	/	220	134	Insomnia Severity Index	/
Jitka Bušková	Cross-sectional	2023	Czech Republic)	30.3±5.3	/	325	213	/	Anxiety、Depression
Jia-Peng Yang	Cross-sectional	2023	China	≥18	/	717	174	Diagnostic and Statistical Manual of Mental Disorder	Depression
Keiko Murakami MPH	Cross-sectional	2022	Japan	32	29-35	17586	6560	Athens Insomnia Scale	/
Juan Wang	Cross-sectional	2022	China	30.3±4.2	20-43	665	179	Pittsburgh Sleep Quality Index	Anxiety、Depression
Jiazhou Wang	Cross-sectional	2021	China	30.25±3.99	19-47	2235	423	Insomnia Severity Index	Anxiety、Depression
Wen-Jing Wang	Cross-sectional	2020	China	35	20-44	436	84	DBAS	Anxiety、Depression
Shiho Umeno	Cross-sectional	2020	Japan	30.9 ± 4.7	/	88	37	Insomnia Severity Index	/
Kızılırmak et al.	Cross-sectional	2012	Turkey	25.2±5.49	15-42	486	254	Women's Health Initiative Insomnia	Depression
Fernández-Alonso	Cross-sectional	2012	Japan	31.0± 7.0	/	370	272	Insomnia Severity Index	/
Randi Liset	Cross-sectional	2021	Norway	30.6±4.0	24-43	61	23	BIS and Insomnia disry	Depression
Nuworza Kugbey	Cross-sectional	2021	Ghana	30.5	26-35	214	90	Insomnia Severity Index	Anxiety、Depression
Magdalena Smyka	Cross-sectional	2020	Poland	28.19	17-44	7207	5556	Self compiled surveys (PSQI, Insomnia Severity Index, Stanford Sleep Questionnaire, and Berlin Questionnaire)	Pressure
Wołyńczyk-Gmaj	Cross-sectional	2017	Poland	30.6 ± 5	/	266	106	Athens Insomnia Scale	/
Dorheim	Cross-sectional	2012	Norway	30.9	/	2,816	1743	Bergen Insomnia Scale	Depression
Anthony M Kendle	Cross-sectional	2022	America	32	15-49	47353875	24625	clinical observation	/
Jennifer N. Felder,	Cross-sectional	2021	America	32.32 ±5.50	/	423	356	Insomnia Severity Index	/
David A. Kalmbach	Cross-sectional	2021	America	30.38±4.96	/	65	35	Insomnia Severity Index	/
Mindell	Cross-sectional	2015	America	/	/	997	217	Insomnia Severity Index	/
Manber	Cross-sectional	2013	America	26.5	/	1,289	219	Women's Health Initiative Insomnia Rating Scale	/
Rannveig S. Osnes	Cohort study	2021	Norway	34	20-48	539	247	Pittsburgh Sleep Quality Index	Depression
Rannveig S. Osnesa	Cohort study	2020	Norway	30.5 ± 4.4	/	530	317	The Bergen Insomnia Scale	Anxiety、Depression
Kiviruusu, O.	Cohort study	2020	Poland	30.7		1,635	948	Basic Nordic Sleep Questionnaire	Anxiety、Depression
David A Kalmbach c	Cohort study	2021	America	28.21±4.32	20-39	39	24	Insomnia Severity Index Pre Sleep Awakening Scale - Cognitive Factors	Depression
Hilla Peltonen	Cohort study	2023	Iran	30.6±4.6	17-48	1414	491	Nordic Sleep Questionnaire	Anxiety、Depression
Nacar and Tashan	Cross-sectional	2019	Turkey	27.7±5.0	18-35	436	157	Women's Health Initiative Insomnia Rating Scale (WHIIRS)	/
Ko	Cohort study	2012	Korea	32.3 ± 3.8	20-45	642	218	Women's Health Initiative Insomnia Rating Scale	/
Cristina Liebana-Presa	Cohort study	2022	Spain	33.61	20-47	297	225	The General Health Questionnaire (GHQ-28)	/
Dolatian	Cohort study	2014	Switzerland	/	18-45	231	119	Insomnia Severity Index	/
Román-Gálvez	Cohort study	2018	Brazil	31.24	/	486	215	Athens Insomnia Scale	/
Madeleine F. Cohen	Cohort study	2022	America	25.36 (5.08)	18-40	600	98	Patient-Reported Outcomes Measurement Information System (PROMIS)	Depression
Ivan D. Sedov & Lianne M. Tomfohr-Madsen	Cohort study	2020	Canada	31.35	>18	142	82	Insomnia Severity Index	Pressure、Depression
Okun and O’Brien	Cohort study	2018	America	29.8b	/	439	146	Insomnia Symptom Questionnaire(ISQ)	/
Facco	Cohort study	2010	America	29.7	/	186	72	Women's Health Initiative Insomnia	/
C. Amezcua-Prieto C.	Radomized controlled trial	2020	Spain	34	18-49	265	159	Athens Insomnia Scale Pittsburgh Sleep Quality Index	/
Rebeca Benito-Villena	Radomized controlled trial	2022	Brazil	27	18-39	270	39	Athens Insomnia Scale	/
David A Kalmbach	Radomized controlled trial	2023	America	28.95±4.51	/	39	9	Pittsburgh Sleep Quality Index Insomnia Severity Index	/
Jennifer N. Felder	Randomized clinical trial	2020	America	33.6±3.7	/	208	110	Pittsburgh Sleep Quality Index	Anxiety、Depression

Meta-analysis was conducted using the comprehensive software STATA 16.0. A random effects model was chosen for analysis based on the level of heterogeneity, typically with I^2^>50%. The I^2^ index was used to assess heterogeneity between point estimates, indicating the proportion of variation between point estimates attributed to heterogeneity. Traditionally, I^2^ values below 25% suggest low heterogeneity, while values between 25% and 50% suggest moderate heterogeneity, and values above 50% suggest high heterogeneity. Subgroup comparison was then employed to further investigate the sources of heterogeneity.

### Quality evaluation

The Newcastle-Ottawa Scale (NOS) was used to evaluate the quality of the included observational studies, and it was generally considered that 1-3 was classified as low quality, 4-6 as medium quality, and 7-9 as high quality. Randomized controlled trials were evaluated using the Cochrane risk bias assessment tool.

## Results

**Figure 1** illustrated the flowchart of the search and selection process. Initially, a search yielded 18,074 records, of which 10,312 were filtered based on title and abstract after removing duplicates. Following a full text review of the remaining 200 studies, 152 studies were excluded due to unclear sleep outcomes. The meta-analysis included 44 studies (9–12, 15–54), involving a total of 47,399,513 participants in the analysis. The literature included 26 cross-sectional studies (9–11, 15–18, 21–26, 28, 30–34, 39, 41, 44, 45, 47, 49, 50), 13 cohort studies (12, 19, 20, 27, 29, 35, 36, 40, 42, 46, 51–53), 4 randomized controlled studies (37, 38, 43, 48), and 1 case-control study (54).

**Figure 2:**
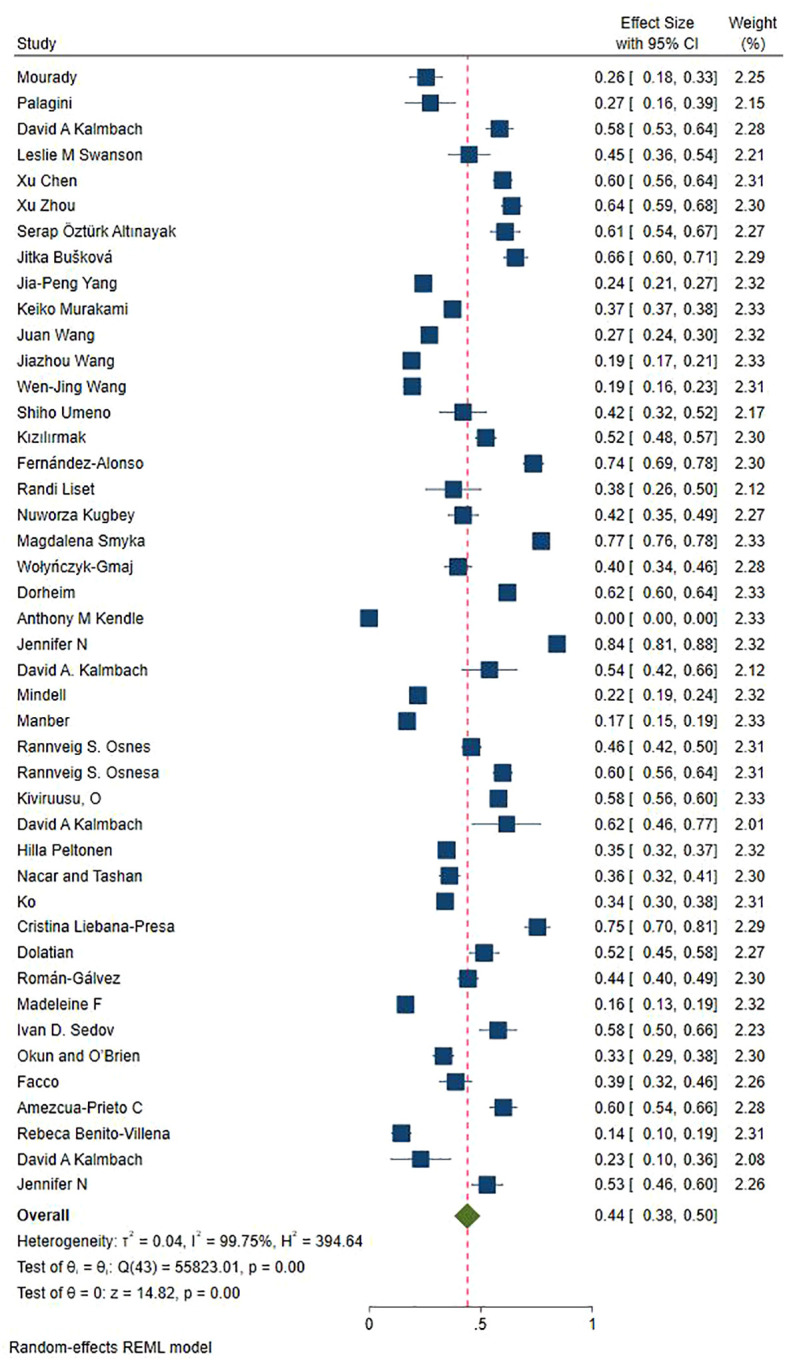
Analysis results of total prevalence of insomnia.

**Figure 3:**
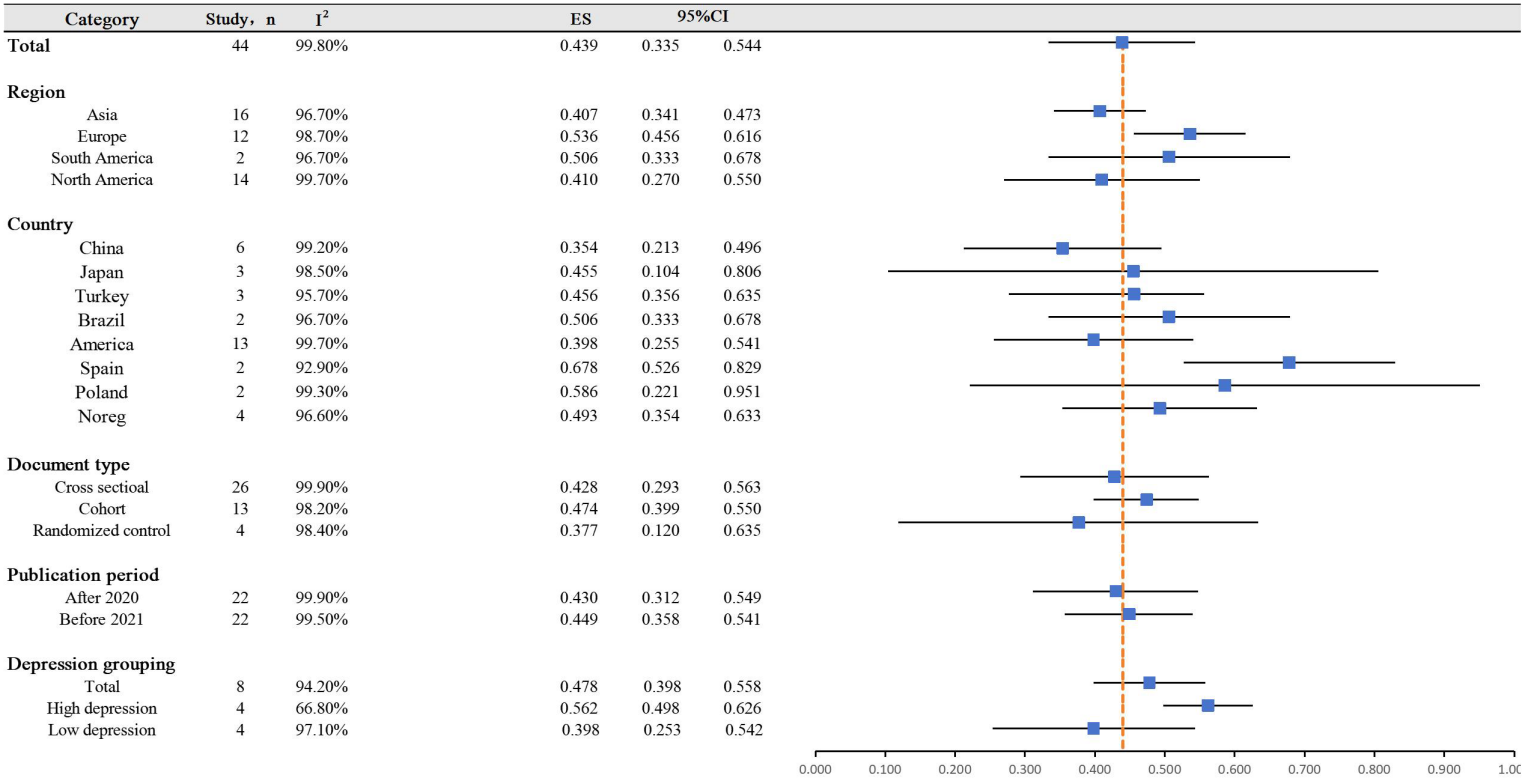
Overall prevalence and subgroup analysis of insomnia.

**Figures 2** and **3** displayed a summary of insomnia prevalence rates and subgroup forest plots, indicating an estimated range of 1% -77.1% for the prevalence of insomnia among 44 study patients. The overall prevalence rate was 43.9% (33.5% - 54.4%). On this basis, multiple subgroup analyses were conducted.

### Subgroup analysis based on region and country

Based on regional grouping results, the prevalence of insomnia in Asia was the lowest (9, 16–18, 23, 24, 29–34, 36, 39, 45, 54), with a specific value of 40.7% (34.1% - 47.3%); Pregnant women in Europe exhibited a high insomnia rate of 53.6% (45.6% - 61.6%) (10, 15, 20–22, 26, 37, 40, 41, 51–53); The prevalence rates in North and South America were 41.0% and 50.6%, respectively (11, 12, 19, 25, 27, 28, 35, 38, 42–44, 46–50). Further analysis of the prevalence of insomnia by the country revealed that Spain has the highest insomnia rate of 67.8% (52.6% - 82.9%, I^2^ = 92.9%) (37, 52), while China has the lowest pregnancy insomnia rate of 35.4% (21.3% -49.6%, I^2^ = 99.2%) (9, 16, 32, 34, 39, 54).

### Subgroup analysis based on study design

According to the analysis of article categories, it was found that the prevalence of insomnia in randomized controlled studies was the lowest at 37.7% (12% - 63.5%) (37, 38, 43, 48).The prevalence of insomnia was relatively similar between cross-sectional studies (9–11, 15–18, 21–26, 28, 30–34, 39, 41, 44, 45, 47, 49, 50) and cohort studies (12, 19, 20, 27, 29, 35, 36, 40, 42, 46, 51–53), at 42.8% and 47.4%, respectively. Based on the analysis of publication years, there was no significant difference in the prevalence of insomnia.

### Subgroup analysis based on psychological depression participants

Based on classification analysis, it was discovered that in studies with a high prevalence of depression, the occurrence of gestational insomnia rose by 56.2% (49.8% - 62.6%). This indicates that depression is a contributing factor to the elevated rates of insomnia.

### Quality evaluation

**Figure 4:**
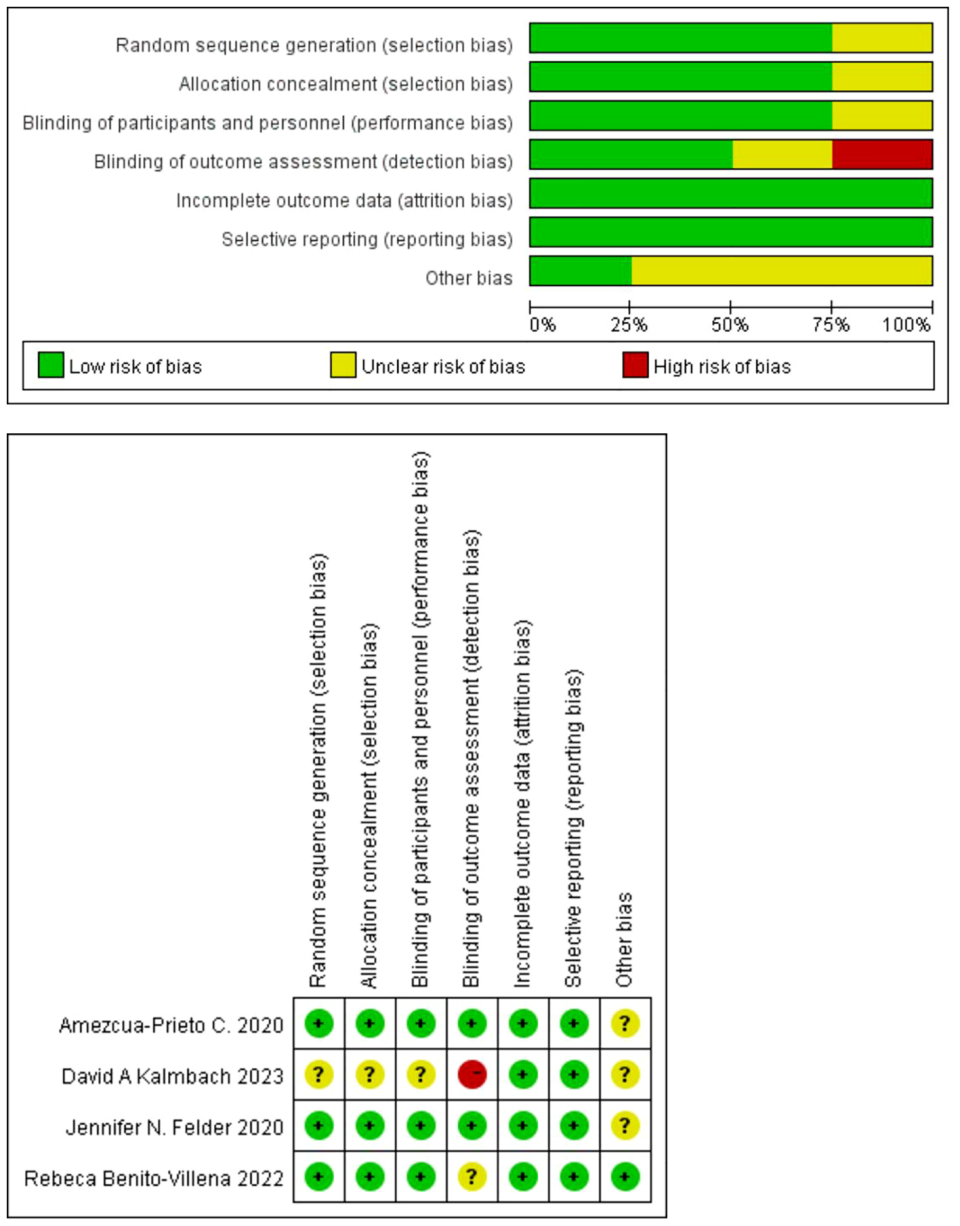
Quality evaluation of 4 randomized controlled trials included.

The overall quality of the included literature was deemed high, with 24 studies in **Table 2** offering moderate evidence and 2 studies presenting low-level evidence. Only one article in the randomized controlled study indicated low quality, as shown in **Figure 4**. The high quality of the papers contributes to the reliability of the analysis results.

**Table 2:** The NOS scales evaluation table for observational studies was included.

Author and year	Research method	Representativeness of the expoaed cohort	Selection of the non exposed cohort	Ascertainment of exposure	Demonstration that outcome of interest was not present at start of study	Comparability of cohorts on the basis of the design or analysis	Assesment of outcome	Was follow-up lonf enough for outcomes to occur	Adequacy of follow up of cohort	Total points
Mourady 2017 (24)	Cross-sectional	⭐	⭐	⭐		⭐⭐	⭐	⭐		7
Palagini 2019 (22)	Cross-sectional	⭐	⭐	⭐		⭐⭐	⭐			6
Kalmbach 2021 (46)	Cross-sectional	⭐	⭐	⭐		⭐	⭐			5
Swanson 2011 (28)	Cross-sectional	⭐	⭐	⭐			⭐			4
Xu 2023 (39)	Cross-sectional	⭐	⭐	⭐		⭐	⭐	⭐	⭐	7
Zhou 2023 (54)	case–control study	⭐	⭐	⭐		⭐⭐	⭐	⭐	⭐	8
Altınayak 2023 (18)	Cross-sectional	⭐	⭐	⭐			⭐	⭐		5
Bušková 2023 (33)	Cross-sectional	⭐	⭐	⭐		⭐	⭐	⭐		6
Yang 2023 (9)	Cross-sectional	⭐	⭐	⭐		⭐	⭐			5
Murakami 2022 (31)	Cross-sectional	⭐	⭐	⭐		⭐⭐	⭐	⭐	⭐	8
Wang 2022 (32)	Cross-sectional	⭐	⭐	⭐	⭐	⭐⭐	⭐	⭐		8
Wang 2021 (34)	Cross-sectional	⭐	⭐	⭐		⭐	⭐			5
Wang 2020 (16)	Cross-sectional	⭐	⭐	⭐	⭐	⭐⭐	⭐	⭐	⭐	9
Umeno 2020 (17)	Cross-sectional	⭐	⭐	⭐		⭐	⭐	⭐		6
Kızılırmak 2012 (30)	Cross-sectional	⭐	⭐	⭐		⭐	⭐	⭐		6
Fernández 2012 (45)	Cross-sectional	⭐	⭐	⭐		⭐	⭐			5
Randi 2021 (21)	Cross-sectional	⭐	⭐	⭐		⭐⭐	⭐	⭐		7
Nuworza 2021 (10)	Cross-sectional	⭐	⭐	⭐		⭐	⭐	⭐	⭐	7
Magdalena 2020 (26)	Cross-sectional	⭐	⭐	⭐		⭐⭐	⭐			6
Wołyńczyk 2017 (15)	Cross-sectional	⭐	⭐	⭐	⭐	⭐⭐	⭐	⭐	⭐	9
Dorheim 2012 (41)	Cross-sectional	⭐	⭐	⭐		⭐⭐	⭐	⭐	⭐	8
Kendle 2022 (50)	Cross-sectional	⭐	⭐	⭐		⭐	⭐			5
Felder 2021 (44)	Cross-sectional	⭐	⭐	⭐		⭐	⭐			5
Kalmbach 2021 (46)	Cross-sectional	⭐	⭐	⭐		⭐	⭐			5
Mindell 2015 (25)	Cross-sectional	⭐	⭐	⭐			⭐			4
Manber 2013 (11)	Cross-sectional	⭐	⭐	⭐		⭐	⭐	⭐		6
Rannveig S 2021 (53)	Cohort study	⭐	⭐	⭐		⭐	⭐	⭐		6
Rannveig S2020 (20)	Cohort study	⭐	⭐	⭐		⭐	⭐	⭐		6
Kiviruusu 2020 (51)	Cohort study	⭐	⭐	⭐		⭐⭐	⭐	⭐	⭐	8
Kalmbach 2021 (46)	Cohort study	⭐	⭐	⭐		⭐	⭐			5
Peltonen 2023 (36)	Cohort study	⭐	⭐	⭐		⭐	⭐	⭐	⭐	7
Nacar and Tashan 2019 (23)	Cross-sectional	⭐	⭐	⭐		⭐⭐	⭐	⭐		7
Ko 2012 (29)	Cohort study	⭐	⭐	⭐		⭐⭐	⭐	⭐	⭐	8
Rebeca 2022 (38)	Cohort study	⭐	⭐	⭐	⭐	⭐	⭐	⭐		7
Dolatian 2014 (40)	Cohort study	⭐	⭐	⭐		⭐				4
Román 2018 (19)	Cohort study	⭐	⭐	⭐		⭐	⭐	⭐		6
Madeleine 2022 (27)	Cohort study	⭐	⭐	⭐		⭐⭐	⭐	⭐	⭐	8
Sedov、Tomfohr-Madsen 2020 (35)	Cohort study	⭐	⭐	⭐		⭐	⭐	⭐		6
Okun and O’Brien 2018 (12)	Cohort study	⭐	⭐	⭐		⭐	⭐			5
Facco 2010 (42)	Cohort study	⭐	⭐	⭐		⭐	⭐	⭐		6

### Sensitivity analysis

**Figure 5:**
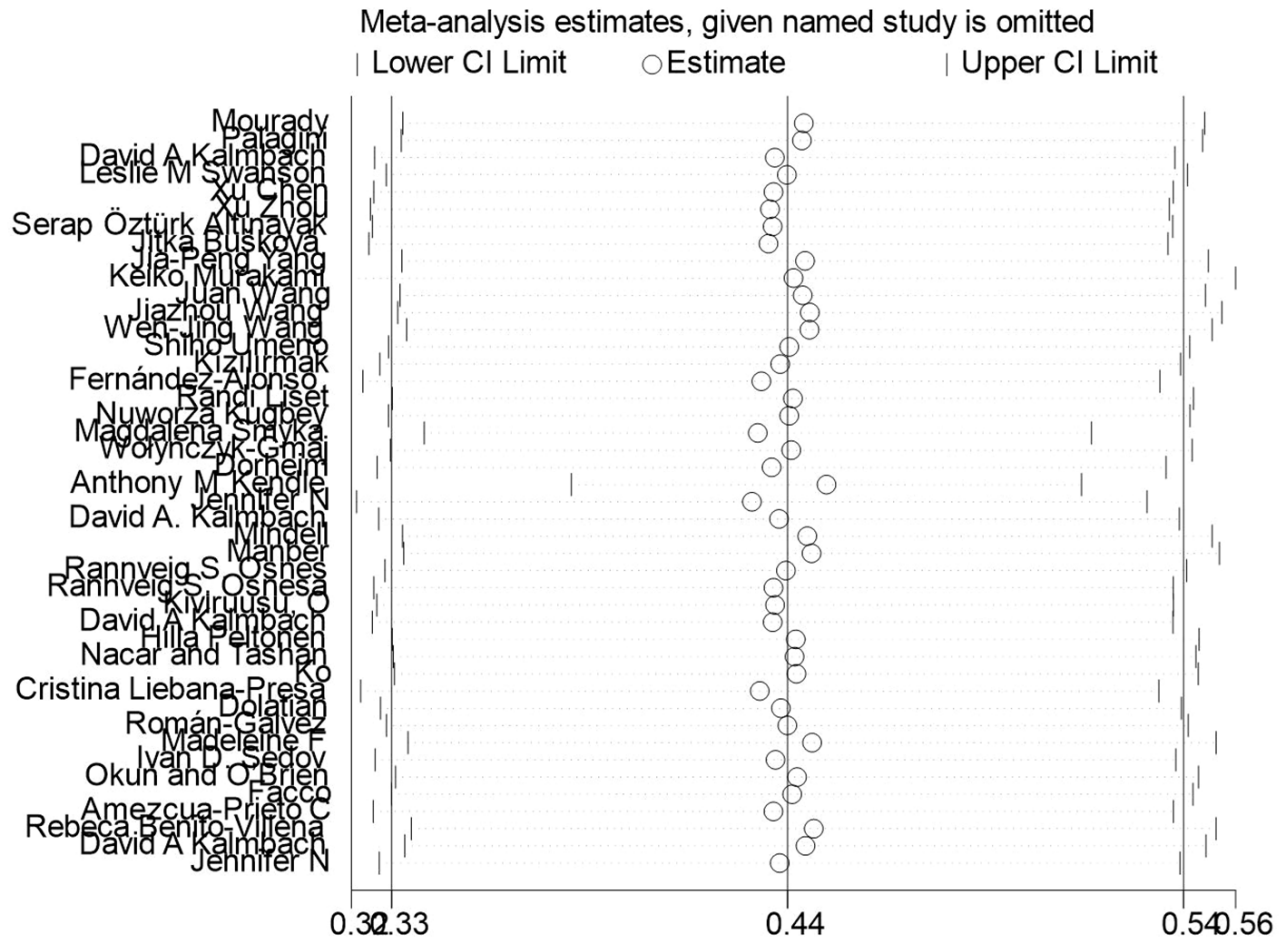
Sensitivity analysis.

After deleting each study item by item (**Figure 5**), the overall estimates remained stable, indicating that the studies did not significantly affect the overall combined prevalence estimate.

## Discussion

An in-depth analysis was conducted on the various factors influencing insomnia during pregnancy, indicating that sleep quality can be significantly impacted by pregnancy. As the pregnancy advances, there is a noticeable decline in sleep quality, with late pregnancy being the most disruptive period (53). Studies have indicated a strong correlation between subjective sleep scores and the severity of depressive symptoms (55). Moreover, pregnancy represents a physiological state characterized by continuous hormonal, physical, and behavioral changes that may significantly alter both sleep quality and duration (56).

The study found that the total prevalence of insomnia during pregnancy was 44.0%, significantly higher than the general population. This highlights the importance of addressing insomnia as a significant health issue during pregnancy. Subgroup analysis by region revealed the highest prevalence in Europe and the lowest in Asia. Further analysis was then conducted at the country level.

Among European countries, Spain, Poland, and Norway exhibited a higher risk of insomnia compared to the overall level of insomnia in this analysis. This finding is in line with previous European studies that have shown a relatively high prevalence of insomnia in the European population (57, 58). Pregnancy events will further exacerbate the burden of female insomnia. A self-reported survey conducted by David O’Regan on individuals with insomnia in Europe identified lifestyle factors and high levels of life stress as the primary causes of insomnia (59). Numerous articles on insomnia have also highlighted the impact of lifestyle factors such as diet, exercise, smoking, and sleep habits on the development of insomnia (60). Additionally, a study on diet and insomnia revealed a positive association between dietary glycemic load and insomnia risk (OR: 1.10; 95% CI, 1.01, 1.20) (61). Spain, Poland, and Norway, being developed countries, often experience higher life burdens and pressures due to the pursuit of a high quality of life. Meanwhile, the preference for sugary foods among Europeans may contribute to the increased risk of insomnia (62–64).

In the Asian subgroup analysis, Japan, a developed country, exhibited a higher prevalence of insomnia during pregnancy, in line with expectations. The study revealed that, apart from economic pressures, the societal focus on women played a significant role in causing insomnia. Notably, China has specific support measures for pregnant women, including reduced working hours, dietary adjustments, and tailored psychological counseling, effectively alleviating psychological stress (65, 66). Moreover, within the Chinese cultural context, pregnant women receive extensive care from family members, contributing to a low insomnia rate (65). These findings offer valuable insights for designing women’s health initiatives. Conversely, the status of women in Japan is comparatively lower, hindering access to social support and contributing to the high prevalence of insomnia among pregnant women (67).

North America mainly includes the United States and Canada. In this analysis, only the United States was considered, revealing a lower insomnia rate compared to the overall level. Throughout the past century, the United States has dedicated efforts to safeguarding women’s power and status. Additionally, being the most developed country globally, the United States boasts top-tier economic and medical advancements. These factors, including a robust system and favorable economic and medical conditions, play a vital role in ensuring a safe pregnancy and reducing the risk of insomnia in pregnant women (68–70).

A correlation between insomnia and depression has been observed. In recent years, the detection rate of pregnancy complications in clinical practice has been on the rise (71), attributed to changes in the living environment, maternal neuroendocrine function, and abnormal fetal development. Studies both domestically and internationally have reported a prevalence rate of depression symptoms during pregnancy among women with pregnancy complications ranging from 29.4% to 39%, significantly surpassing that of healthy pregnant women (72, 73).

Through data analysis, it was found that individuals with high levels of depression were at a higher risk of insomnia, and depression was positively correlated with an increase in sleep latency. A study found differences in the consistency of local activity in the auxiliary motor area and insula between patients with insomnia and those without. Patients with insomnia and severe depression exhibited differences in the intensity of spontaneous activity in the middle frontal gyrus and paracentral lobules compared to those without insomnia. Previous studies have shown that the potential neurobiological mechanisms of depression and insomnia symptoms may have included: (1) abnormalities in monoamine neurotransmitters, especially changes in 5-HT concentration, which were closely related to sleep awakening and depression, such as shortened REM latency in patients with depression; (2) Overexpression of biological clock genes and stress response genes; (3) Dysfunction of the hypothalamic pituitary adrenal axis (HPA) and abnormal release of cortisol (74). According to data, there was a close relationship between insomnia during pregnancy and depression at both the symptom and disease levels. Some women’s insomnia symptoms were relatively stable in the early stages of pregnancy, but temporarily increased in the late stages of pregnancy. This is closely associated with significant physiological and psychological changes, and pregnancy can be characterized as a period of heightened biological and situational stress (75), which may activate latent vulnerabilities and magnify them. Hence, many pregnant women might experience a worsening of insomnia symptoms in the later stages of pregnancy. For instance, around 70% of individuals with depression experience symptoms of insomnia, and the prevalence of depression among pregnant women with insomnia is 3-4 times higher than in those without insomnia (76). This bidirectional and cumulative relationship necessitates greater clinical attention, as gestational insomnia and depression both pose risk factors for adverse pregnancy outcomes.

Insomnia during pregnancy is not inherently harmful, but it can contribute to an elevated risk of various health complications for women, such as stillbirth, miscarriage, perinatal depression, and other adverse outcomes. As a result, it is essential to focus on non-pharmacological methods for preventing and managing insomnia during pregnancy. Psychological and social factors play a significant role in the varying prevalence of insomnia during pregnancy, with psychological factors often being linked to some social factors. Pan Chen and Eric S Kim suggest that enhancing overall well-being can be an effective way to alleviate negative psychological symptoms (77, 78). Therefore, enhancing the focus on women during pregnancy is crucial for safeguarding their health (79). China, with its historical cultural background, has shown a greater emphasis on women’s health compared to other developed countries. Drawing from China’s approach, strategies such as reducing the work intensity of pregnant women, promoting increased attention from family members and social groups, providing regular psychological counseling, and encouraging appropriate exercise like relaxation training and mindfulness can help alleviate psychological issues during pregnancy and improve sleep quality to some extent (80).

## Limitations

In this meta-analysis, significant heterogeneity was found, which may be attributed to population characteristics, study design, evaluation of insomnia and measurement of outcomes, as well as clinical stages of pregnancy. Secondly, insomnia mainly came from subjective reports or questionnaire surveys, and differences in diagnosis may have a certain impact on the results. Due to the fact that the summary result of single arm rate is a descriptive result and not a difference comparison result, the statistical significance of publication bias is not strong. We strictly followed the inclusion and exclusion criteria to manually screen relevant articles, without any restrictions on the language or year of the study, thus minimizing the possibility of omitting any research related to the topic; We also conducted a stratified analysis based on geographical location, publication time, literature type, and degree of depression. Therefore, compared to other small-scale studies, our research results may have more reference value and robustness. Finally, due to the limited data on insomnia across various gestational periods, a subgroup analysis based on these different periods has not yet been conducted. Further studies are anticipated to provide additional verification in the future.

## Conclusion

The prevalence of insomnia during pregnancy, reaching as high as 44%, has been displaying an upward trend year by year. Urgent attention must be directed toward women’s health issues. Insomnia during pregnancy not only elevates the risk of adverse pregnancy outcomes for women but also significantly affects fetal development and postpartum well-being. As widely acknowledged, insomnia during pregnancy stems from various complex factors, with regional disparities emerging as a central aspect warranting special attention. We aim to undertake further regional research in the future to enhance clinical evidence for developing regional policies aimed at safeguarding women’s health.

## Data Availability

The original contributions presented in the study are included in the article/**Supplementary Material**. Further inquiries can be directed to the corresponding authors.
