# Supplementary material for: Evaluating the global prevalence of insomnia during pregnancy through standardized questionnaires and diagnostic criteria: a systematic review and meta-analysis
Source: Front Psychiatry. 2024 Aug 13;15:1427255. doi: 10.3389/fpsyt.2024.1427255 (PMC11348333; doi:10.3389/fpsyt.2024.1427255)
Supplement: Supplementary file 1 [file DataSheet1.zip › Supplementary Material 2.docx]

**Supplemental Table 1**: Medical subject headings (MeSH) and non-MeSH terms used to search relevant publications on the relation between Evaluating the Global Incidence of Insomnia During Pregnancy Through Standardized Questionnaires and Diagnostic Criteria: A Systematic Review and Meta-Analysis ^1^

| **Database** | **Step** | **Terms** | **Results** |
| --- | --- | --- | --- |
| **PubMed** | 1 | "Pregnancy" [Mesh] OR "Pregnancy"[Title/Abstract] OR "Pregnan*"[Title/Abstract] OR "Pregnancies"[Title/Abstract] OR "gestation"[Title/Abstract] | 1,211,585 |
|  | 2 | "Sleep Initiation and Maintenance Disorders" [Mesh] OR "Sleep Initiation and Maintenance Disorders"[Title/Abstract] OR "DIMS"[Title/Abstract] OR "Disorders of Initiating and Maintaining Sleep"[Title/Abstract] OR "Insomnia Disorder"[Title/Abstract] OR "Insomnia"[Title/Abstract] OR "Insomnias"[Title/Abstract] OR "Sleeplessness"[Title/Abstract] OR "Chronic Insomnia"[Title/Abstract] OR "Nonorganic Insomnia"[Title/Abstract] OR "Early Awakening"[Title/Abstract] OR "Primary Insomnia"[Title/Abstract] OR "Psychophysiological Insomnia"[Title/Abstract] OR "sleep"[Title/Abstract] OR "sleep disturbance"[Title/Abstract] OR "sleep disturbances"[Title/Abstract] OR "sleep problem"[Title/Abstract] OR "sleep problems"[Title/Abstract] OR "sleep quality"[Title/Abstract] OR "Sleep Initiation"[Title/Abstract] | 247,594 |
|  | 3 | 1781/01/01:2024/02/29[Date - Publication] |  |
|  | 4 | #1 AND #2 AND #3 | **5617** |
| **Embase** | 1 | 'Pregnancy'/exp OR 'Pregnancy':ab,ti,kw OR 'Pregnan*':ab,ti,kw OR 'Pregnancies':ab,ti,kw OR 'gestation':ab,ti,kw | 1,273,110 |
|  | 2 | 'Insomnia'/exp OR 'Sleep Initiation and Maintenance Disorders':ab,ti,kw OR 'DIMS':ab,ti,kw OR 'Disorders of Initiating and Maintaining Sleep':ab,ti,kw OR 'Insomnia Disorder':ab,ti,kw OR 'Insomnia':ab,ti,kw OR 'Insomnias':ab,ti,kw OR 'Sleeplessness':ab,ti,kw OR 'Chronic Insomnia':ab,ti,kw OR 'Nonorganic Insomnia':ab,ti,kw OR 'Early Awakening':ab,ti,kw OR 'Primary Insomnia':ab,ti,kw OR 'Psychophysiological Insomnia':ab,ti,kw OR 'sleep':ab,ti,kw OR 'sleep disturbance':ab,ti,kw OR 'sleep disturbances':ab,ti,kw OR 'sleep problem':ab,ti,kw OR 'sleep problems':ab,ti,kw OR 'sleep quality':ab,ti,kw OR 'Sleep Initiation':ab,ti,kw | 418,548 |
|  | 3 | [01-01-1966]/sd NOT [01-03-2024]/sd AND [<1966-2024]/py |  |
|  | 4 | #1 AND #2 AND #3 | **8919** |
| **Web of Science** | 1 | TS=("Pregnancy" OR "Pregnan*" OR "Pregnancies" OR "gestation") | 354,275 |
|  | 2 | TS=("Sleep Initiation and Maintenance Disorders" OR "DIMS" OR "Disorders of Initiating and Maintaining Sleep" OR "Insomnia Disorder" OR "Insomnia" OR "Insomnias" OR "Sleeplessness" OR "Chronic Insomnia" OR "Nonorganic Insomnia" OR "Early Awakening" OR "Primary Insomnia" OR "Psychophysiological Insomnia" OR "sleep" OR "sleep disturbance" OR "sleep disturbances" OR "sleep problem" OR "sleep problems" OR "sleep quality" OR "Sleep Initiation") | 194,315 |
|  | 3 | DOP=(1997-01-01/2024-02-29) |  |
|  | 4 | #1 AND #2 AND #3 | **3,538** |
| **Total** |  |  |  |

^1^ Two investigators (ZSP & JYJ) searched the online databases independently.
